# Fighting the global counterfeit medicines challenge: A consumer-facing communication strategy in the US is an imperative

**DOI:** 10.7189/jogh.12.03018

**Published:** 2022-04-23

**Authors:** Sylvester Senyo Ofori-Parku

**Affiliations:** School of Journalism and Communication, University of Oregon, Eugene, Oregon, USA

The headline of a January 18, 2022 news story in the *Wall Street Journal* read “*Drugmaker Gilead Alleges Counterfeiting Ring Sold Its HIV Drugs.*” The article reported that counterfeit HIV medicines sometimes contained over-the-counter painkillers or antipsychotic drugs. Unfortunately, the subject in this title is not a one-off occurrence; it is a global health issue. Counterfeit medicine trafficking is one of the world’s fastest-growing criminal enterprises [1]. Analysts estimate the global counterfeit market to be worth between US$200 and US$432 billion [1-3]. These figures make pharmaceuticals the number one illicit activity, ahead of other underground economic activities such as prostitution, human trafficking, marijuana, electronics, and arms sales [3]. This article observes that medicine counterfeiting is as big a challenge for the industrialized world as it is for low-income countries. Supply-side tactics concentrating on rules, law enforcement, and technology are commonplace solutions. However, a complementary consumer-focused approach beyond awareness creation that draws on decision science on counterfeit product/medicine usage can accomplish far more.

## DEFINING COUNTERFEIT MEDICINES

There is no universally accepted definition of counterfeit medicines. However, the World Health Organisation (WHO) uses labels like “substandard”, “falsely labelled”, “falsified”, and “counterfeit” to characterize medicines that are forged to seem genuine [4]. The term is not synonymous with low-cost generics. The latter is just as safe and effective as existing brand-name versions protected by intellectual property. Counterfeits, however, may contain no active ingredients, incorrect amounts, or incorrect ingredients (eg, chalk, mercury, paint, deadly poisons). They may also include significant impurities and contaminants, generic or branded, and claim to cure life-threatening conditions like cancer, or routine ones. These include geographically-specific medications such as antimalarials or antibiotics [4,5] that may be either cheap or expensive. They are available via illegal street markets, websites, legitimate pharmacies, clinics, and hospitals [6]. This health and safety challenge is fueled by corruption in governments, the proliferation of illicit online pharmacies that often falsely portray themselves as Canadian, complex medical products supply chains, the availability of technologies for counterfeiting and packaging products, lack of access to medical care, and consumers’ appetite for cheap medicines [2,3,7].

**Figure:**
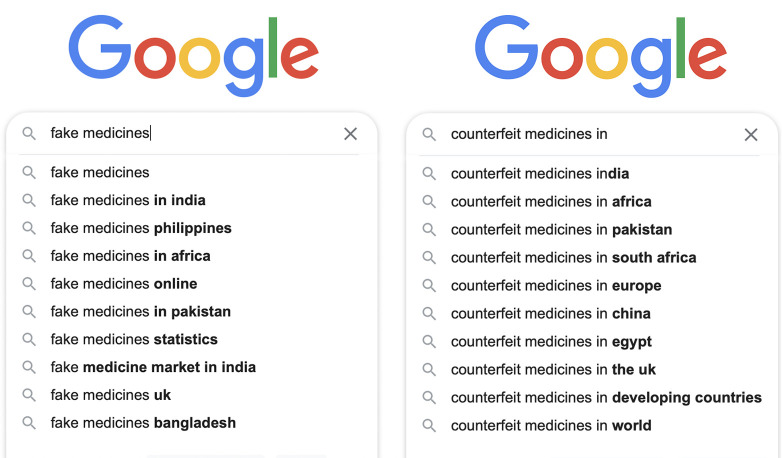
Photo: Searching for “fake medicines” and “counterfeit medicines” in Google return search results that predominantly associate the counterfeit medicine challenge with low-income countries. Source: images from Google search screenshot.

## NOT JUST A “DEVELOPING” WORLD PROBLEM

This problem is often framed as a third-world concern even by the WHO [4,5] and online searches show a similar perspective (**Figure 1**), but it is a truly global challenge involving both richer and poorer regions of the world. The Pharmaceutical Security Institute’s data on incidents of counterfeiting show that illicit trafficking of medicines in 137 countries increased by 38%, from 3146 in 2016 to 4344 in 2020 [8]. The 2020 data involves 2451 different drugs across all therapeutic categories. North America ranked number one among regions with the highest incidents of counterfeit medicine seizures in 2020 (32%), followed by Asia Pacific (23%), Latin America, Eurasia, Near East, Europe, and Africa (3%), in that order.

While only 4% of randomly sampled online pharmacies (out of 11 700) adhere to US pharmacy laws and practice standards, nearly 25% of adults have purchased prescriptions online [6]. Moreover, 20% of the consumers made such purchases via websites that have no link with local pharmacies or health insurance plans [6]. A whopping 96% of the 35 000 active online pharmacies (selling prescriptions medicines to US consumers) operating in 2019 did not comply with applicable legal and pharmacy standards [7]. Thus, much of fake medicine consumption in the US thrives via fraudulent online pharmacies.

## IMPLICATIONS FOR PUBLIC HEALTH

Counterfeit medicine proliferation is a more significant public health threat than diseases they purport to cure [9]. Those containing no active ingredients may be just as dangerous as those containing contaminants. At best, such medications do not treat the ailments they purport to treat. At worse, they contain toxic ingredients that outright kill patients. In both cases, counterfeit medications not only present brand safety and economic challenges for pharmaceutical companies [2,3], but they also present serious global public health and safety consequences. Fake medicines have dire long-term health consequences for consumers (eg, antimicrobial resistance, organ failure, drug-resistant infections, overdose, or even death) [4,5,8]. At the systemic level, counterfeit medicines are an invisible, less understood barrier to equitable access to health, and are “more insidious than drug prices” [9].

## THE CHALLENGE

Despite counterfeit medicines’ health and safety risks, public awareness of the prevalence and consequences of taking such drugs is lacking [9]. Lack of rigorous and universal drug regulations, the complex nature of the global pharmaceuticals supply chains, and the sophistication of counterfeit medicine packaging are among the factors that make it difficult for regulators, pharmaceutical firms, activists, and consumers to curtail the problem [2,3]. Except for a few studies in low-income countries [5,9,10], studies and regulators focus on supply-side factors, laws and regulations, and anti-counterfeiting technologies [4]. Like all counterfeit goods, price is a critical driver of fake medicine consumption – especially in low-income or rural contexts. It is a tall order for laws and technology to succeed when there is a ready market or appetite for counterfeit goods. Besides, for some consumer segments (eg, young and middle-income earners), the price may not be a motivation for consuming counterfeits [10]. To curtail the global counterfeit medicines market and ensure public health and safety, advocates must complement supply-side efforts with consumer-facing informational campaigns that focus on attitude change [10,11]. Some ongoing efforts include the WHO’s webpage on “substandard and falsified medical products;” US Food and Drug Administration’s *Know Your Source, Filled with Empty Promises,* and *BeSafeRx;* Alliance for Safe Online Pharmacies’ *Buy SafeRx*; and Pfizer’s *Fight the Fakes.*

However, a consumer-facing approach is not simply a matter of creating awareness, as counterfeit medicine campaigns often entail. Insight into the psycho-social factors that underlie consumer attitudes, risk perceptions, purchase intentions, and fake medicine consumption is critical. Applying these insights would help improve communication outcomes. The science of consumer attitudes and decision-making about counterfeit medicines/goods provides us with the tools necessary to implement evidence-based demand-side communication strategies that address the problem in the US and internationally.

## SOME STRATEGIC RECOMMENDATIONS

Some argue that, unlike other consumer products, medicines do not confer social status or prestige. As a result, consumers are more receptive to low-cost counterfeit alternatives, believing or hoping that they are as effective as the often more expensive genuine options [10,12]. Granted this, the increasing availability of low-cost generics (eg, 9/10 prescriptions in the US are generic drugs [13]) partly fulfils the need for cheaper alternatives. Besides, for some consumer segments, paying less for medicines is not as much about the price as it is about the *feeling* that they are “getting even” with large pharmaceutical companies [4]. Furthermore, people take medicines primarily in the hope of becoming better. Hence, functionality may be more critical for consuming medications than other consumer goods. Yet, counterfeit medicine consumption has similar social desirability or (negative) prestige avoidance effects as other consumer products. For example, individuals who believe their significant others (eg, family and friends) would disapprove of their use of counterfeit medicines are more likely to have negative attitudes toward counterfeit drugs, heightened risk perceptions, and low purchasing intentions [5,11]. Therefore, a social desirability effect exists among fake medicine consumers. This evidence, combined with the enormous contribution of online pharmacies in the US to counterfeit medicine supply and consumption [6], points to the persuasive prospects of public health campaigns that emphasize the social desirability of *not* “buying medicines from sketchy online pharmacies.” In other words, appealing to consumers’ need for social approval by their significant others would influence their attitudes, risk perceptions, and counterfeit medicines consumption.

Second, another evidence-based communication appeal is rooted in the ethics of consuming counterfeit medicines. Some consumer segments do not want to patronize counterfeits for moral reasons: they do not want to condone illegal activities. Similar effects exist for general counterfeit goods [10], as well as medicine [11,12]. Interestingly, even if they believe counterfeit items are as effective as (or less expensive than) genuine ones, this consumer segment does not prefer them. This insight implies that letting this consumer segment know which pharmacies sell counterfeit medicines and using ethics-based message appeals would influence desirable decision outcomes. Like social desirability appeals effects, ethical considerations drive attitudes and behaviour intentions and shape risk perceptions.

Third, a consequence of not addressing the demand-side of the counterfeit medicine challenge is that it can turn into a kind of treadmill that fuels future consumption. This is especially true if an individual does not experience any adverse health effects from consuming counterfeit drugs. Typically, having a negative experience with a product would make one less likely to patronize similar products at a future date. But sometimes, having a prior experience with counterfeit medicines is associated with positive attitudes, behavioural intentions, and reduced risk perceptions [11]. The placebo effect may explain this [14], and this effect does not necessarily go away even when patients know they consumed a fake [15]. Thus, since not all counterfeits are the same, individuals who (*un)knowingly* consume substandard medicines (eg, containing less or no active ingredients) may get better. Getting well reinforces their efficacy beliefs about such treatment options, even if they become aware that they are fakes. This phenomenon is truer for injections and creams than it is for pills [15].

## CONCLUSION

Counterfeit medicines are an enormous global health challenge that cannot be simply curtailed through laws and regulations, drug seizures, or anti-counterfeiting technologies. The US is a significant market for counterfeit medicines, and addressing the demand-side of the challenge is critical. There is an urgent need to execute anti-counterfeit medicines campaigns with tailored messages that tap into consumers’ ethical norms, social beliefs, and safety needs.
